# Understanding and safely using ophthalmic lasers

**Published:** 2015

**Authors:** Ismael Cordero

**Affiliations:** Clinical Engineer, Philadelphia, USA. **ismaelcordero@me.com**

**Figure:**
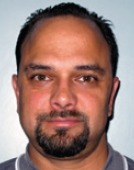
Ismael Cordero

Ophthalmic lasers allow precise treatment of a range of eye problems with little risk of infection. Many laser procedures are relatively pain free and can be performed on an outpatient basis. The combination of safety, accuracy, and relative low cost make lasers very useful ophthalmic tools.

The word laser is an acronym for ‘**l**ight **a**mplification by **s**timulated **e**mission of **r**adiation’. Laser light is coherent (the waves are in phase in space and time), monochromatic (just one colour or wavelength), and collimated (light is emitted as a narrow beam in a specific direction). Laser beams are produced by the excitation of atoms to a higher than usual energy state. Laser light (radiation) is emitted as the atoms return to their original energy levels.

The main components of a laser system are the laser console, the foot pedal, and the laser delivery system. Different delivery systems, connected to the console by a fibre optic cable, can be used to transmit the laser energy to the patient's eye (Figure 1): an endoprobe (a small fibre optic probe that is inserted into the eye), a slit lamp, an operating microscope, oran indirect ophthalmoscope.

**‘Lasers essentially destroy tissue in order to have a beneficial effect on the eye’**

Different types of lasers emit specific wavelengths of light and are used to treat specific eye problems. Lasers are commonly named according to the active material used. For instance, an argon laser contains argon gas as its active material, whereas the YAG laser contains a solid material made up of yttrium, aluminium, and garnet.

The effects that lasers have on eye tissues are both a function of the molecular composition of the tissue and of the wavelength and power of the laser light. Lasers essentially destroy tissue in order to have a beneficial effect on the eye.

The **argon** laser emits blue-green wavelengths, which are absorbed by the cells under the retina and by the red haemoglobin in blood. These blue-green wavelengths can pass through the fluid inside the eye without causing damage. For this reason, the argon laser is used extensively in the treatment of diabetic retinopathy. The argon laser can burn and seal the leaking blood vessels, also known as photocoagulation.

**Figure 1.**
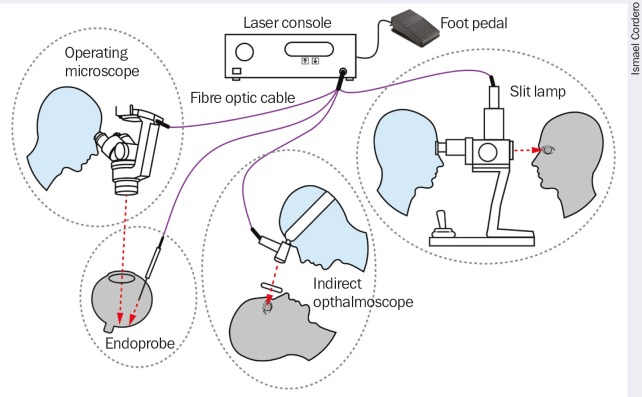


Retinal detachment is another serious eye problem that can be treated using an argon laser. The laser is used to weld the detached retina to the underlying choroid layer of the eye. Some forms of glaucoma may also be treated with argon lasers. For instance, angle-closure glaucoma can be treated by using an argon laser to create a tiny hole in the iris (a capsulotomy), which allows excess fluid inside the eye to drain to reduce pressure.

Macular degeneration is sometimes treated with an argon or **krypton** laser. In this treatment, the laser is used to destroy abnormal blood vessels so that haemorrhage or scarring will not damage central vision.

The **YAG** 1064 nm infrared laser generates short-pulsed, high-energy light beams to cut, perforate, or fragment tissue. For patients that develop posterior capsular opacification after receiving cataract surgery, the YAG laser is commonly used to vaporise a portion of the capsule, allowing light to fully reach the retina. A frequency-doubled YAG green laser (wavelength 532 nm) can also be used to create a capsulotomy to treat angle-closure glaucoma, producing similar results to that of an argon laser.

The **diode** laser has similar applications to both the argon and the YAG laser. The advantage of diode lasers is that they are much smaller and portable, produce less heat, and require much less maintenance than other types of lasers.

Lasers units also include a red pointer or target laser beam, which causes no harm to the tissue, to enable the surgeon to see where the treatment laser shots will land.

## Using lasers safely

To ensure safe operation and prevent hazards and unintended exposure to laser beams you must follow protective measures:

- To prevent unwanted exposure to laser energy, always review and observe the safety precautions outlined in the operator manuals before using the device.

**Figure 3.**
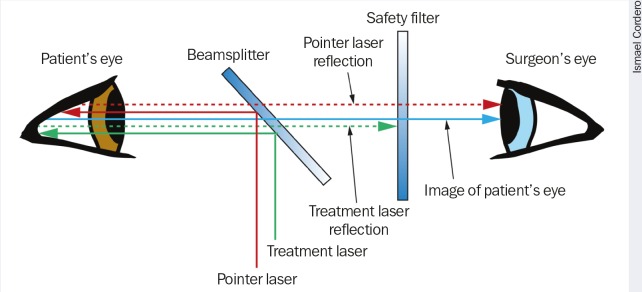

**Figure 2.**
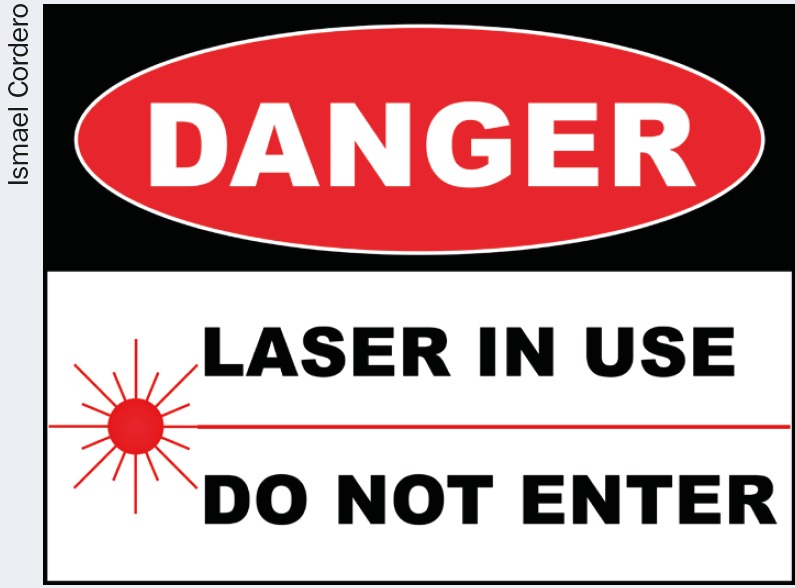
Warning sign
- The laser device should only be used by a qualified physician.
- Do not use any laser device if you think it is not functioning properly.
- Do not bend or stretch the fibre cords. When storing or packing, roll them into large circles to prevent damaging the fibres.
- All maintenance and calibrations on laser devices should only be conducted by factory-trained technicians.
- When conducting laser treatments, place a sign on the door to the treatment area to alert people that laser therapy is taking place and to not enter (Figure 2).
- Laser beams reflected from reflective surfaces can harm your eyes, the patient's eyes, or others' eyes. Any mirror or metal object that reflects the laser beam can constitute a reflection hazard. Be sure to remove all reflection hazards near the laser. Use non-reflecting instruments whenever possible. Be careful not to direct the laser beam at unintended objects.
- All personnel in the treatment area should wear the proper laser safety glasses designed to filter the specific wavelengths and power of the laser being used. The safety eyewear needed is based on the Maximum Permissible Exposure (MPE), Nominal Ocular Hazard Area (NOHA), and Nominal Ocular Hazard Distance (NOHD) for each of the delivery devices used with the laser system, as well as the configuration of the treatment room. For additional information, refer to the laser device's user manual and well as international laser standards and guidelines.
- Safety filters (Figure 3) protect the physician from back-scattered laser light. Integral eye safety filters should be permanently installed in every slit lamp and laser indirect ophthalmoscope. For endophotocoagulation or for operating microscope use, a separate discrete eye safety filter assembly must be installed into each viewing path of the operating microscope. All eye safety filters have an optical density (OD) at the laser wavelength sufficient to permit long-term viewing of diffuse laser light.

